# Antioxidant and Lipoxygenase Inhibitory Activities of Essential Oils from Endemic Plants of Côte d’Ivoire: *Zanthoxylum mezoneurispinosum* Ake Assi and *Zanthoxylum psammophilum* Ake Assi

**DOI:** 10.3390/molecules24132445

**Published:** 2019-07-03

**Authors:** Evelyne A. Tanoh, Fatimata Nea, Tierry Kenne Kemene, Manon Genva, Matthew Saive, Felix Z. Tonzibo, Marie-Laure Fauconnier

**Affiliations:** 1 Laboratory of Biological Organic Chemistry, UFR-SSMT, University Felix Houphouet-Boigny, 01 BP 582 Abidjan 01, Côte d’Ivoire; 2 Laboratory of Chemistry of Natural Molecules, University of Liège, Gembloux Agro-Bio Tech, 2, Passage of Deportés, 5030 Gembloux, Belgium

**Keywords:** *Zanthoxylum mezoneurispinosum*, *Zanthoxylum psammophilum*, essential oil, undecan-2-one, tridecan-2-one, antioxidant activity, DPPH, FRAP, lipoxygenase inhibitory activity

## Abstract

*Zanthoxylum mezoneurispinosum* Ake Assi and *Zanthoxylum psammophilum* Ake Assi are species endemic to Côte d’Ivoire. In this study, we determined, for the first time, the composition and biological activities of essential oils obtained from each of these plants. Essential oils were obtained by hydrodistillation from different organs of each plant with a Clevenger-type apparatus and analyzed by gas chromatography–mass spectrometry (GC-MS). Thirty-four components, accounting for more than 99.9% of the overall composition, were identified in the oils. The *Z. psammophilum* leaf and trunk bark oils exhibited two unusual methylketones, undecan-2-one and tridecan-2-one, whereas the root oil was rich in thymol and sesquiterpenoids. The *Z. mezoneurispinosum* leaf and trunk bark oils were rich in monoterpenoids, whereas sesquiterpenoids were predominant in the root oil. These samples produced, for the first time, some new chemical profiles of essential oils. The oils’ antioxidant activities were determined using 2,2-diphenyl-1-picrylhydrazyl (DPPH) radical scavenging capacity and ferric reducing antioxidant power (FRAP) assays. The results showed that the essential oil isolated from roots of *Z. mezoneurispinosum* had the highest antioxidant activity, which is in accordance with the high thymol content of that oil. We also determined the lipoxygenase inhibitory activities of the essential oils. The results showed that all of the tested oils displayed high and close lipoxygenase inhibitory activities.

## 1. Introduction

The genus *Zanthoxylum,* family of Rutaceae, contains approximately 250 species that are distributed in subtropical and tropical climates in America, Africa, Asia, and Australia [1]. This genus is well-known for its interesting biological properties, including antioxidant, antimicrobial, antifungal, and anticancer properties [2,3,4,5,6,7]. These properties help to explain the extensive use of these plants in traditional medicine to treat a range of diseases, including anemia, cancer, sickle cell disease, infertility, rheumatism, osteoarthritis, and dysentery [8]. Furthermore, the fruits that species of the genus *Zanthoxylum* produce are consumed as food condiments in China [9,10].

**Figure 1:**
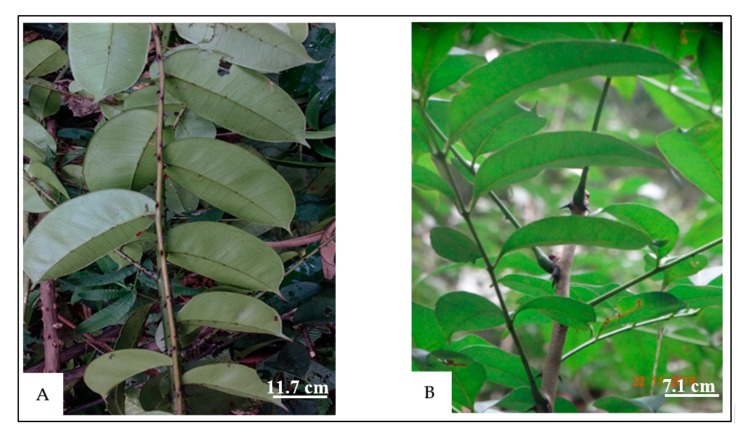
Leaves of Zanthoxylum psammophilum (**A**) and Zanthoxylum mezoneurispinosum (**B**).

*Zanthoxylum psammophilum* and *Zanthoxylum mezoneurispinosum* are two species of the genus *Zanthoxylum* that are endemic to Côte d’Ivoire [11]. *Z. psammophilum* is a thin, hairless lianascent plant that grows up to 15 m tall and 2 cm in diameter with solitary stem spines. *Z. psammophilum* presents a thick trunk bark that is easy to separate from the bark (the separation occurs at the vascular cambium level). Its leaves are composed of five to seven pairs of alternate leaflets with a moderately thorny petiole (Figure 1A). *Z. mezoneurispinosum* is a sarmentary shrub that grows to 10–15 m tall with twin stem spines. The trunk bark is not very thick and is quite difficult to collect. The spines of the relatively large, leafy shoots are black and measure 0.5–1 cm. They are located on either side of the petiole, with the tip bent downwards (Figure 1B). The nonvolatile compounds that the roots of these two species produce have already been studied. Two new benzophenanthridines, including 8-methoxy-7,8-dihydrofagaridine, were isolated from *Z. psammophilum* roots. Benzophenanthridines are known to have a wide range of interesting properties, including antibacterial, antifungal, and anticancer activities [12]. A cycloheptapeptide, akeassimezorine, was also found in *Z. mezoneurispinosum* roots [1]. To date, the volatile molecules of these two species have not been studied, either for their chemical composition or for their biological activities. The aim of this work is to isolate essential oils from the leaves, trunk bark, and roots of these two species, characterize the oils by GC-MS, and evaluate their antioxidant and lipoxygenase inhibitory activities.

## 2. Results

### 2.1. Yields

**Figure 2:**
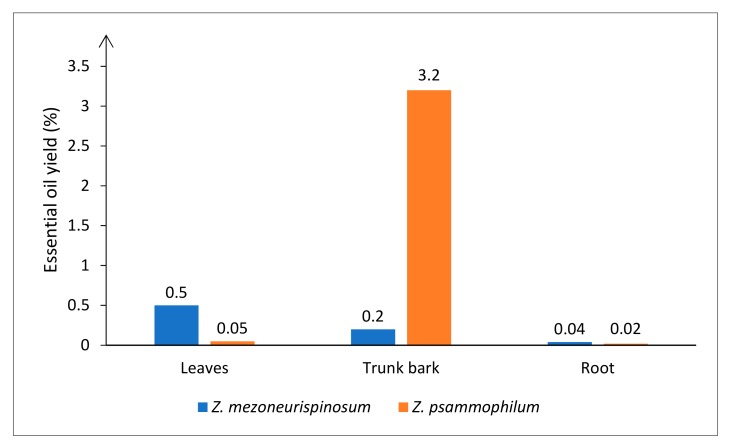
Essential oil hydrodistillation yields (%) from *Z. psammophilum* and *Z. mezoneurispinosum* organs. Yields are expressed as g/100 g of fresh material.

Different hydrodistillation yields were obtained depending on the species and on which fresh organ was hydrodistilled. Average yields of 0.5% and 0.05% for leaf oil, 0.2% and 3.2% for trunk bark oil, and 0.04% and 0.02% for root oil were obtained for *Z. mezoneurispinosum* and *Z. psammophilum,* respectively (Figure 2). Moreover, the obtained essential oils differed by their smells. For both species, leaves had a pronounced herbal scent, and roots had a spice-like scent. Nevertheless, the scent of the trunk bark differed between species. The *Z. mezoneurispinosum* trunk bark oil had a wood-like scent, whereas the *Z. psammophilum* trunk bark oil had a buttery note.

### 2.2. Chemical Composition of Essential Oils

#### 2.2.1. *Zanthoxylum psammophilum*

Several compounds were identified in the essential oils isolated from organs of *Z. psammophilum*. Sixteen molecules were found in the leaf oil, four in the trunk bark oil, and 24 in the root oil. These compounds represent more than 99.9% of each essential oil’s overall composition (Table 1). The leaf oil and the trunk bark oil consisted mainly of nonterpenic acyclic compounds (82.2% and >99.9%, respectively). The root oil was characterized by a high content of sesquiterpenes (75.3%).

Leaves: A highly diverse set of molecules was found in the essential oil isolated from *Z. psammophilum* leaves (Table 1). Methylketones (79.4%) were the main compounds found in this oil; however, sesquiterpenes (14.9%) and diterpenes (2.9%) were also present. The leaf oil’s major components were found to be tridecan-2-one (54.4%), undecan-2-one (20.6%), pentadecan-2-one (4.4%), and tridecan-2-ol (3.8%).

Trunk bark: The essential oil isolated from *Z. psammophilum* trunk bark was mainly composed of methylketones, such as undecan-2-one (61.0%) and tridecan-2-one (37.1%). Other compounds, such as undecan-2-ol and tridecan-2-ol, were also found in this oil but in much lower quantities (1.5% and 0.4%, respectively) (Table 1).

Roots: As shown in Table 1, sesquiterpenes (75.3%), monoterpenes (18.2%), and methyl ketones (6.6%) were dominant in the essential oil isolated from *Z. psammophilum* roots. The main components were found to be β-caryophyllene (21.8%), thymol (15.7%), α-humulene (13.6%), elemol (5.2%), α-copaene (5.7%), β-selinene (4.9%), caryophyllene oxide (4.7%), δ-cadinene (4.5%), and cyclosativene (4.2%).

#### 2.2.2. *Zanthoxylum mezoneurispinosum*

The analysis of the chemical composition of essential oils isolated from *Z. mezoneurispinosum* organs revealed 15 compounds for the leaves, 19 compounds for the trunk bark, and 27 compounds for the roots. These compounds represent more than 99.9% of each essential oil’s overall composition (Table 1). The essential oils isolated from the trunk bark and from the leaves were predominantly composed of monoterpenes (83.3% and 92.1%, respectively). Sesquiterpenes represented 16.7% and 7.9% of the composition of the trunk bark oil and the leaf oil, respectively. The essential oil isolated from the roots was mainly composed of sesquiterpenes (81.5%) and monoterpenes (18.6%).

Leaves: The essential oil isolated from *Z. mezoneurispinosum* leaves was rich in monoterpenes, including α-pinene (55.4%), sabinene (10.1%), (*E*)-β-ocimene (8.9%), β-myrcene (4.5%), linalool (3.8%), and terpineol (3.2%). β-caryophyllene (3.0%) was the main sesquiterpene found in this essential oil.

Trunk bark: The essential oil isolated from *Z. mezoneurispinosum* trunk bark was rich in monoterpenes, including α-pinene (50.9%), sabinene (9.7%), linalool (5.1%), terpineol (4.2%), and β-myrcene (3.2%). β-caryophyllene (2.5%) and γ-eudesmol (5.2%) were the main sesquiterpenes found in this essential oil.

Roots: The main compounds that were present in the essential oil isolated from *Z. mezoneurispinosum* roots included γ-elemene (24.8%), cyclosativene (11.9%), viridiflorol (7.1%), α-copaene (5.4%), and elemol (5.0%). β-selinene (4.5%) and γ-eudesmol (4.3%) were the main sesquiterpenes and α-pinene (11.0%) was the main monoterpene.

### 2.3. Biological Activities of the Essential Oils

#### 2.3.1. Antioxidant Activity

The in vitro antioxidant activities of the essential oils isolated from the two *Zanthoxylum* species were evaluated in two different ways. The 2,2-diphenyl-1-picrylhydrazyl (DPPH) free radical test was used to evaluate the H-donating or radical scavenging ability of the oils. The ferric reducing antioxidant power (FRAP) assay was used to estimate the ferric-reducing capacity of the oils. For each tested essential oil and each assay, the concentration ranging from 100 to 25 µg/mL that caused a 50% inhibition of the oxidant activity (IC_50_) was determined and compared to a standard. A low IC_50_ value indicates a high antioxidant activity.

##### The DPPH Free Radical Scavenging Method

**Figure 3:**
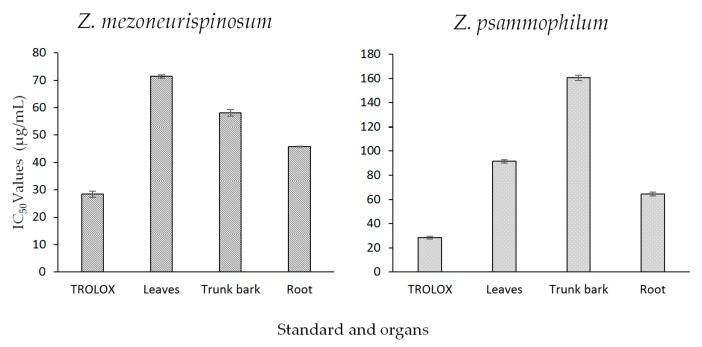
The IC_50_ values (µg/mL), obtained with the 2,2-diphenyl-1-picrylhydrazyl (DPPH) assay, of essential oils isolated from different organs of *Z. mezoneurispinosum* (**A**) and *Z. psammophilum* (**B**). Data are expressed as the mean and standard value, *n* = 3.

The antioxidant activities of essential oils isolated from different organs of both *Zanthoxylum* species were evaluated with the DPPH assay. Trolox, a molecule well-known for its antioxidant properties, was used as a standard. Figure 3 shows the results. The IC_50_ values of the essential oils varied by species and organ. For both plants, an increase in essential oil concentration induced a higher antioxidant activity (*p*-value <0.05). Moreover, at the same concentration and for the same organ, all essential oils isolated from *Z. psammophilum* had higher antioxidant activities than essential oils isolated from *Z. mezoneurispinosum* (*p*-value <0.001). For the *Z. mezoneurispinosum* specie, the essential oil isolated from the roots showed the highest antioxidant activity, with an IC_50_ value of 45.8 ± 0.1 µg/mL. The Trolox standard showed an IC_50_ value of 28.4 ± 1.1 µg/mL. The essential oils isolated from the trunk bark and leaves had IC_50_ values of 58.1 ± 1.2 µg/mL and 71.4 ± 0.5 µg/mL, respectively. The essential oils isolated from *Z. psammophilum* exhibited higher IC_50_ values: 64.5 ± 1.5 µg/mL for the roots, 91.5 ± 1.4 µg/mL for the leaves, and 160.5 ± 2.1 µg/mL for the trunk bark.

##### Ferric Reducing Antioxidant Power (FRAP) Assay

**Figure 4:**
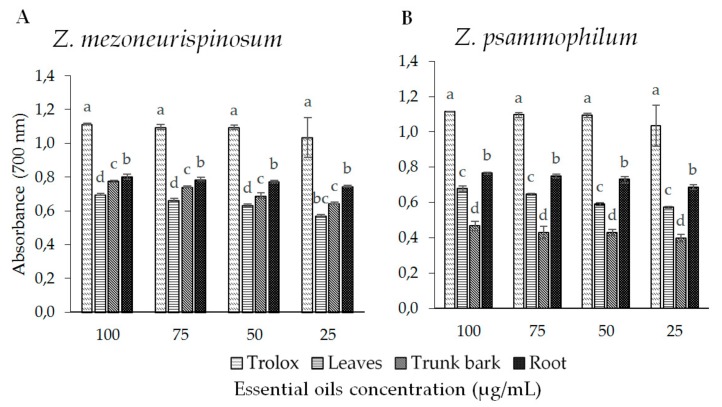
The ferric-reducing power of essential oils isolated from different organs of *Z. mezoneurispinosum* (**A**) and *Z. psammophilum* (**B**). Data are expressed as the mean and standard value, *n* = 3. Within a concentration, mean values followed by the same letter are not significantly different according to Tukey’s test (*p* < 0.001).

The antioxidant activity of the essential oils was also evaluated using the reducing power test. The presence of reducing agents in oils causes a reduction of Fe^3+^, which is a complex in ferrous form. Therefore, we evaluated Fe^2+^ by measuring and monitoring the increase in blue color density in reaction media of essential oils of *Z. mezoneurispinosum* and *Z. psammophilum*. Figure 4A shows the results for the *Z. mezoneurispinosum* essential oil; Figure 4B shows the results for the *Z. psammophilum* essential oil. For each essential oil and each species, a dose effect was observed as a significant increase in antioxidant activity, which was found to be correlated with an increase in concentration (*p*-value <0.001). Moreover, all of the essential oils isolated from *Z. mezoneurispinosum* had higher antioxidant activities (*p*-value <0.001) than those isolated from *Z. psammophilum*.

#### 2.3.2. Lipoxygenase Inhibitory Activity

The lipoxygenase inhibitory activities of essential oils isolated from different organs of *Z. mezoneurispinosum* and *Z. psammophilum* were evaluated by measuring the ability of each oil to inhibit the activity of lipoxygenase, which is an enzyme involved in the inflammation process. Quercetin was used as the reference standard. The IC_50_ values are presented in Table 2. The results showed that the essential oils isolated from different organs of *Z. mezoneurispinosum* had lower IC_50_ values than those isolated from different organs of *Z. psammophilum* (*p*-value <0.05). For all essential oils and both plant species, dose effects were observed as higher essential oil concentrations always resulted in greater lipoxygenase inhibitory activities (*p*-value <0.05).

## 3. Discussion

### 3.1. Essential Oil Yields

In this study, essential oils were isolated from different organs of *Z. psammophilum* and *Z. mezoneurispinosum*, two plant species endemic to Côte d’Ivoire [13]. For several other *Zanthoxylum* species, the literature reports essential oil hydrodistillation yields that range from 0.05 to 1.90% (*w/w*) [14,15,16]. The yields of essential oils isolated from leaves and roots of *Z. psammophilum* (0.05% and 0.02%, *w/w*), and from leaves, trunk bark, and roots of *Z. mezoneurispinosum* (0.50%, 0.2% and 0.04%, *w/w*), that were obtained in the present study seem, therefore, to be consistent with those reported in the literature. The yield value of essential oil of 3.2% (*w/w*) that was obtained from the trunk bark of *Z. psammophilum* seems to be high; however, it is similar to the yield obtained from dried fruits of another *Zanthoxylum* species (*Zanthoxylum leprieurii* from Cameroon) [3] and can be explained by the morphology of the trunk bark of this specie presenting a thick bark.

### 3.2. Chemical Composition of the Essential Oils

This work represents the first report regarding the characterization of the chemical composition of the essential oils isolated from the different organs of *Zanthoxylum* species. Methylketones were found as main dominant chemical component of the essential oils isolated from aerial parts of *Z. psammophilum*. This is consistent with the chemical composition of essential oils isolated from the leaves and trunk bark of *Z. leprieurii* from Côte d’Ivoire [15]. Moreover, in Indian *Z. armatum* and Australian *Z. pinnatum*, methylketones have also been reported as major components of leaves essential oils (undecan-2-one (54.3%) and tridecan-2-one (31.7%)) for the former and undecan-2-one (46.0%) and tridecan-2-one (27.1%) for the latter [13,14,17]. However, when comparing the methylketones proportion in these two species with the ones in *Z. psammophilum*, it appeared that tridecan-2-one compound is the most dominant in *Z. psammophilum* leaves whereas in the trunk bark, it was the undecan-2-one which was the prevailing molecule. This could suggest that the leaves and trunk bark of *Z. psammophilum* could exhibit interesting properties as they are used for their flavor and fragrance in the food, pharmaceutical, and perfumery industries [18]. In combination with undecan-2-one, tridecan-2-one also have well-known antibacterial and insecticidal activities [19,20]. The composition of the essential oil isolated from *Z. psammophilum* roots was different to those isolated from the aerial parts of the plant, as β-caryophyllene (21.8%), α-humulene (13.6%), and thymol (15.7%) were the main compounds in the root oil. The high proportion of β-caryophyllene in this oil is interesting because this molecule is known to have several biological activities, including good antioxidant properties [21].

Monoterpenoids dominated the chemical composition of the essential oils isolated from aerial parts of *Z. mezoneurispinosum*. This is in agreement with the chemical composition of several other *Zanthoxylum* species [14,22,23]. Moreover, two new compounds, (*E*)-β-ocimene and γ-eudesmol, which, to our knowledge, have not been described before in these species, were present in their essential oils. These two molecules have interesting antioxidant, antibacterial, and antimicrobial properties [24,25]. Additionally, the essential oil isolated from *Z. mezoneurispinosum* roots was found to be rich in sesquiterpenoids, as high quantities of cyclosativene (11.9%) were present in this oil. This is an interesting finding, as it appears that this molecule has only been found before in trace quantities in leaf essential oils obtained from *Xylopia aromatica* and *Persea americanum*, which are grown in Cuba [26]. To our knowledge, this is the first time that high quantities of this compound have been described in an essential oil. The high content of cyclosativene is of consequence for the valorization of this essential oil as this molecule has strong antimicrobial, antioxidant, and insecticidal properties [27,28,29,30]. Moreover, γ-elemene (24.8%), which is a molecule known for its insecticidal, antimicrobial, and antioxidant properties, was reported in high quantities in essential oil isolated from *Z. mezoneurispinosum* roots [31,32].

The complete chemical characterization of essential oils isolated from organs of *Z. psammophilum* and *Z. mezoneurispinosum* allows us to highlight the potential of these two plants as a source of therapeutic molecules for use in traditional medicine. We found compounds with antibacterial, antimicrobial, and antioxidant activities in the different essential oils that we isolated. Moreover, other applications might be considered for some of the newly described essential oils. Some could, in fact, be used as a new source of raw material for the flavour industry or as a new bioactive agent in pest management.

### 3.3. Biological Properties of the Essential Oils

A wide range of molecules with interesting biological properties were identified in the essential oils produced from the different organs of the two *Zanthoxylum* species. Reports on the interesting biological properties of solvant extracts and essential oils from a range of *Zanthoxylum* species can also be found in the literature [33,34]. For instance, *Zanthoxylum limonella* and *Z. leprieurii* were shown to have antioxidant activities, and *Z. armatum* was shown to possess anti-inflammatory properties [35]. It would seem then plausible to reveal antioxidant and anti-inflammatory properties of the essential oils that were isolated in this paper.

Several analytical methods have been developed to determine the antioxidant capacity of natural substances in vitro. Here, the DPPH radical scavenging and FRAP methods were used to evaluate the antioxidant activity of the essential oils. The two tests showed that the essential oils isolated from *Z. mezoneurispinosum* have a better antioxidant activity than those isolated from *Z. psammophilum*, probably due to their different chemical composition. The weak antioxidant activity of the *Z. psammophilum* essential oils may be due to their high non-terpenic compound content. The essential oils isolated from *Z. mezoneurispinozum* were found to be rich in monoterpenes, which are molecules known for their high antioxidant activity [17]. Moreover, the essential oil isolated from *Z. mezoneurispinosum* roots contained a high proportion of thymol, which is a molecule that is known to have antioxidant properties [36]. The presence of this compound could explain why the antioxidant activity of the essential oil isolated from *Z. mezoneurispinosum* roots was higher than that isolated from *Z. psammophilum*.

Regarding anti-inflammatory activity, we investigated the lipoxygenase inhibitory properties of the isolated essential oils. The results showed that all of the tested essential oils have strong lipoxygenase inhibitory activity due to the presence of β-caryophyllene, α-pinene and methylketones. These compounds are well-known for their anti-inflammatory activity [37,38,39,40]. Our results are congruent with [41] who highlighted that other *Zanthoxylum* species, *Z. capense*, has good anti-inflammatory properties. 

However, in vitro antioxidant and anti-inflammatory interesting results obtained in this study should be confirmed by in vivo assays before considering the utilization of those essential oils in human care [42].

## 4. Material and Methods

### 4.1. Plant Materials and Hydrodistillation Procedure

Various organs of *Z. psammophilum* and *Z. mezoneurispinosum*, including leaves, trunk bark, and roots, were collected in southeastern Côte d’Ivoire, namely Agboville (5°55′40″ N, 4°12′47″ W) and Grand Lahou (5°15′2.4″ N, 5°0′12″ W), in October and November 2017. The plant material was authenticated by botanists at the National Flora Center (CNF) (Abidjan, Côte d’Ivoire) and the vegetation improvement laboratory of Nangui Abrogoua University (Côte d’Ivoire). A specimen of each species (AA21002 and AA21009 for *Z. psammophilum* and *Z. mezoneurispinosum*, respectively) was deposited at the Herbarium of the CNF. The scent of the essential oils was determined by five trained panelists. Fresh material (1.0–1.5 kg) was hydrodistillated for 3 h using a Clevenger-type apparatus. Essential oils were collected, dried over anhydrous Na_2_SO_4_, stored in sealed amber vials, and kept under refrigeration (4–6 °C) until analysis. The essential oil hydrodistillation yield was determined as the ratio of the mass of oil after distillation to the mass of fresh organs.

### 4.2. Essential Oil Analyses

Ten milligrams of essential oil was dissolved in 100 mL of hexane and analyzed by GC-MS (Agilent, Santa Clara, CA, USA). For each essential oil, this manipulation was repeated three times.

GC-MS was carried out using an Agilent GC system 7890B (Agilent, Santa Clara, CA, USA) fitted with a split-splitless injector and coupled to an Agilent MSD 5977B detector. One microliter of 0.01% essential oil solution was injected, and the analytical conditions were fixed as follows: injection mode: splitless at 300 °C; HP-5MS capillary column (Agilent, Santa Clara, CA, USA) (30 m × 0.25 mm, df = 0.25 µm); temperature program: from 50 °C (1 min) to 300 °C (5 min) at a rate of 5 °C/min. The carrier gas was helium at a flow rate of 1.2 mL/min. The mass spectra were recorded in Electron Ionization mode at 70 eV (scanned mass range: 40–400 *m/z*). The source and quadrupole temperatures were fixed at 230 °C and 150 °C, respectively. The component identification was performed on the basis of chromatographic retention indices (RI) and by comparison of the recorded spectra with a computed data library (Pal 600K®) [43]. RI values were measured on an HP-5MS column (Agilent, Santa Clara, CA, USA). RI calculations were performed in temperature program mode according to [44,45,46,47,48]; a mixture of homologues n-alkanes (C7–C30) was used under the same chromatographic conditions. The main components were confirmed by comparison of their retention and MS spectrum data with co-injected pure references (Sigma, Darmstadt, Germany) when commercially available.

### 4.3. Biological Activities

#### 4.3.1. Antioxidant Activity

The antioxidant activities of essential oils produced from *Zanthoxylum* species were evaluated using in vitro DPPH and FRAP assays.

##### 2,2-diphenyl-1-picrylhydrazyl Radical Scavenging Capacity

The free radical scavenging activity [43] was determined in triplicate according to [48] with slight modifications in concentrations [49,50]. Briefly, 0.5 mL of the essential oils at four concentrations (25, 50, 75, and 100 µg/mL) in methanol was added to 1 mL of a stable solution of DPPH (0.06 mM, methanol) [50]. The mixture was vortexed for about 1 min and then incubated at room temperature in the dark for 30 min. Absorbance was measured at 517 nm in a spectrophotometer Ultrospec 7000 UV–visible, dual beam spectrophotometer (GE Healthcare, Cambridge, UK). Trolox (Sigma, Darmstadt, Germany) was used as a standard (a positive control) under the same conditions. All tests were performed in triplicate. The scavenging activity of essential oils on DPPH radicals was calculated as DPPH (%) inhibition using the following equation:

% Inhibition = ((Ab − Aa)/Ab) × 100(1)

Ab = absorbance of the blank sample.

Aa = absorbance of the test sample.

##### Ferric-Reducing Power Determination

The reducing power of essential oils and the standard (Trolox) was evaluated as described by Singleton and Hseu [51,52]. In brief, a 1 mL sample at four concentrations (25, 50, 75, and 100 µg/mL) (in triplicate for each concentration) and prepared in methanol was mixed with phosphate buffer (1 mL, 0.2 M, pH = 6.6) and 1% potassium ferricyanide [K_3_Fe(CN)_6_] solution (1 mL) and incubated at 50 °C for 20 min. Afterwards, trichloroacetic acid (TCA) (1 mL, 10% *v/v*) was added to the solution, which was then centrifuged for 10 min at 3000 *g*. The recovered supernatant was mixed with distilled water (1.5 mL) and 0.1% *v/v* FeCl_3_ (150 µL). The absorbance of the resulting solution was then measured at 700 nm [53]. An increase in absorbance (when compared to the blank) indicates an increase in the reducing power. Absorbance due to the essential oils themselves (at the different concentrations) was systematically subtracted for each assay.

#### 4.3.2. Lipoxygenase Inhibitory Activity

The in vitro lipoxygenase inhibitory activity of essential oils was determined by measuring the inhibition power of essential oils on lipoxygenase activity (EC 1.13.11.12). The spectrophotometric method described by Lyckander and Malterud (1992) was used with some minor modifications. Briefly, a reaction mixture containing essential oils in various concentrations (100, 75, 50, and 25 µg/mL of methanol) (in triplicate for each concentration), lipoxygenase (Sigma, Darmstadt, Germany), and 35 µL (0.1 mg/mL) of a 0.2 M borate buffer solution (pH = 9.0) was incubated for 15 min at 25 °C. The reaction was then initiated by addition of 35 µL of a substrate solution (linoleic acid 250 µM) and the absorbance was measured at 234 nm. Quercetin (Sigma) was used as a standard inhibitor at the same concentration as the essential oils [54,55,56]. The inhibition percentage of lipoxygenase activity was calculated as follows:

Inhibition percentage (%) = 100 × (OD_blank_ − OD_sample_)/OD_blank_ (2)

where OD_blank_ is the Optical Density (OD) of the reaction media without the essential oil, and OD_sample_ is the OD of the reaction media with the essential oil minus the OD value of the diluted essential oil (to compensate for absorbance due to the essential oils themselves).

The amount of DPPH that was inhibited by the essential oils and the lipoxygenase inhibitory activity of these essential oils were expressed as the percent concentration corresponding to a 50% inhibition (IC_50_).

### 4.4. Statistical Analysis

The DPPH and FRAP methods were used to determine the antioxidant activity of the essential oils. The measurements to determine the antioxidant activity as well as the lipoxygenase inhibitory activity were performed in triplicate. Two factors were considered for the statistical analysis: the organ and the concentration. Experimental data are expressed as mean ± SD. The analysis of variance between the different averages was carried out by ANOVA using the Minitab 18 software (Pennsylvania, PA, USA). The significance level for the tests was set at 5%. The structuring of averages was done using Dunnett’s test. A multiple comparison analysis was performed using Tukey’s test on both DPPH and lipoxygenase inhibitory activities.

## 5. Conclusions

The aim of this study was to evaluate the potential of *Z. psammophilum* and *Z. mezoneurispinosum*, two species endemic to Côte d’Ivoire, as a source of therapeutic molecules for use in traditional medicine or other applications. The results showed that the essential oils isolated from these plants contain interesting molecules and display some good and previously unknown biological properties. Although the essential oils exhibited only moderate antioxidant activities, all isolated oils displayed elevated lipoxygenase inhibitory activities. Essential oils isolated from these plants could, therefore, be used in traditional medicine, given this interesting property. However, subsequent studies on these oils should take into account the plant’s age and the harvesting season to better understand their impact on the oils’ composition and biological properties. Moreover, the chemical characterization of these oils led to the identification of molecules that potentially have other biological activities, such as antimicrobial properties. Hence, it could be interesting to test the antimicrobial activity of these essential oils as plant extracts are increasingly being used in therapeutics to control multi-resistant organisms [57,58]. The use of these newly described essential oils would, of course, require an extensive study of their cytotoxicity before application in human or animal care. The insecticidal activity of these essential oils could also represent another potential source of valorization, as the plant protection sector is always searching for new bio-based active compounds to replace conventional insecticides. Perfumers are also avid for new scents for their creations, which may constitute a high added-value valorization for these newly described essential oils.

However, it is important to mention that both plants are endemic and, currently, non-cultivated. If local populations begin to use them for their properties or in other applications, preservation initiatives (e.g., plant nurseries) will be crucial to prevent them from disappearing.

## Figures and Tables

**Table 1:** The chemical composition of essential oils isolated from different organs of two *Zanthoxylum* species endemic to Côte d’Ivoire. Data are expressed as the mean of triplicates.

N°	Compounds	Cas Number	Identification	RI^a^	RI^b^	*Zanthoxylum mezoneurispinosum*			*Zanthoxylum psammophilum*		
						Leaves	Trunk Bark	Root	Leaves	Trunk Bark	Root
1	α-pinene	80-56-8	MS, RI, STD	931	933	55.4 ± 0.9	50.9 ± 3.6	11.0 ± 0.5	–	–	–
2	sabinene	3387-41-5	MS, RI, STD	969	971	10.1 ± 0.1	9.7 ± 0.4	0.4 ± 0.0	–	–	–
3	β-pinene	18,172-67-3	MS, RI, STD	970	974	–	–	0.7 ± 0.0	–	–	–
4	β-myrcene	123-35-3	MS, RI, STD	987	989	4.5 ± 0.1	3.2 ± 0.5	1.1 ± 0.0	–	–	–
5	α-phellandrene	99-83-2	MS, RI, STD	1000	1002	1.6 ± 0.5	tr	–	–	–	–
6	p-cymene	25,155-15-1	MS, RI, STD	1022	1023	2.5 ± 0.1	2.6 ± 0.5	tr	–	–	–
7	limonene	138-86-3	MS, RI, STD	1023	1027	tr	tr	1.4 ± 0.1	–	–	–
8	eucalyptol	470-82-6	MS, RI, STD	1026	1029	–	–	1.6 ± 0.1	–	–	–
9	(*E*)-β-ocimene	13,877-91-3	MS, RI, STD	1041	1046	8.9 ± 0.2	1.7 ± 0.1	0.7 ± 0.1	–	–	–
10	linalool	78-70-6	MS, RI, STD	1094	1098	3.8 ± 0.2	5.1 ± 1.0	0.3 ± 0.0	–	–	tr
11	undecane	1120-21-4	MS, RI, STD	1100	1099	–	–	–	1.1 ± 0.1	tr	–
12	alloocimene	7216-56-0	MS, RI	1125	1129	2.3 ± 0.1	2.1 ± 0.2	0.8 ± 0.1	–	–	–
13	4-terpineol	562-74-3	MS, RI, STD	1177	1178	tr	0.7 ± 0.2	tr	tr	tr	0.4 ± 0.0
14	4-isopropylcyclohexen-2-one	500-02-7	MS, RI	1184	1186	–	3.2 ± 0.9	–	–	–	–
15	terpineol	98-55-5	MS, RI, STD	1190	1190	3.2 ± 0.1	4.2 ± 0.6	tr	tr	tr	tr
16	citronellol	106-22-9	MS, RI, STD	1225	1227	–	–	–	–	tr	1.0 ± 0.0
17	geraniol	106-24-1	MS, RI, STD	1250	1254	–	–	tr	–	tr	1.0 ± 0.0
18	thymol	89-83-8	MS, RI, STD	1287	1291	tr	tr	0.7 ± 0.2	tr	tr	15.7 ± 0.2
19	undecan-2-one	112-12-9	MS, RI, STD	1289	1293	–	–	–	20.6 ± 0.1	61.0 ± 0.4	3.3 ± 0.2
20	undecan-2-ol	1653-30-1	MS, RI, STD	1298	1300	–	–	–	2.3 ± 0.1	1.5 ± 0.2	tr
21	α-cubebene	17,699-14-8	MS, RI	1350	1353	–	tr	0.4 ± 0.0	–	–	–
22	cyclosativene	22,469-52-9	MS, RI	1368	1373	–	–	11.9 ± 0.2	–	–	4.2 ± 0.1
23	copaene	3856-25-5	MS, RI, STD	1376	1379	–	tr	5.4 ± 0.1	–	tr	5.7 ± 0.0
24	α-bergamotene	17,699-05-7	MS, RI, STD	1411	1417	–	–	–	0.7 ± 0.0	–	–
25	α-gurjunene	489-40-7	MS, RI	1412	1419	–	–	–	0.8 ± 0.0	–	–
26	**β-caryophyllene**	87-44-5	MS, RI, STD	1419	1423	3.0 ± 0.1	2.5 ± 0.1	tr	1.7 ± 0.0	tr	**21.8 ± 0.1**
27	cadina-4(14),5-diene	54,324-03-7	MS, RI	1430	1434	0.7 ± 0.0	tr	2.5 ± 0.1	–	–	–
28	**γ-elemene**	3242-08-8	MS, RI	1440	1444	tr	1.6 ± 0.1	**24.8 ± 0.3**	tr	tr	0.8 ± 0.2
29	**α-humulene**	6753-98-6	MS, RI, STD	1456	1458	1.4 ± 0.1	1.2 ± 0.1	tr	1.1 ± 0.1	tr	**13.6 ± 0.2**
30	alloaromadendrene	24,246-27-9	MS, RI	1457	1466	–	–	–	–	–	1.55 ± 0.1
31	germacrene D	23,986-74-5	MS, RI, STD	1482	1486	0.7 ± 0.0	tr	2.4 ± 0.2	–	–	–
32	β-selinene	17,066-67-0	MS, RI	1488	1493	tr	tr	4.5 ± 0.2	tr	tr	4.9 ± 0.1
33	**tridecan-2-one**	593-08-8	MS, RI, STD	1490	1495	tr	tr	tr	**54.4 ± 0.4**	37.1 ± 0.3	3.3 ± 0.1
34	selina-4(14),7(11)-diene	515-17-3	MS, RI	1495	1498	tr	0.8 ± 0.3	0.9 ± 0.0	tr	tr	2.7 ± 0.1
35	tridecan-2-ol	1653-31-2	MS, RI, STD	1495	1501	–	–	–	3.8 ± 0.0	0.4 ± 0.0	tr
36	γ-cadinene	39,029-41-9	MS, RI	1513	1517	–	–	–	tr	tr	1.0 ± 0.1
37	δ-cadinene	483-76-1	MS, RI	1524	1527	1.5 ± 0.1	1.3 ± 0.1	3.7 ± 0.2	0.8 ± 0.0	tr	4.5 ± 0.1
38	**elemol**	639-99-6	MS, RI, STD	1547	1552	tr	1.7 ± 0.5	5.0 ± 0.3	1.4 ± 0.0	tr	**5.2 ± 0.0**
39	nerolidol	7212-44-4	MS, RI, STD	1557	1564	–	–	–	1.68 ± 0.05	tr	0.4 ± 0.01
40	sphathulenol	6750-60-3	MS, RI	1578	1584	0.7 ± 0.03	1.0 ± 0.2	2.5 ± 0.0	tr	–	0.5 ± 0.1
41	caryophyllene oxide	1139-30-6	MS, RI, STD	1583	1588	–	–	–	1.1 ± 0.0	tr	4.7 ± 0.2
42	guaiol	489-86-1	MS, RI	1600	1604	–	–	1.6 ± 0.2	–	–	–
43	**viridiflorol**	552-02-3	MS, RI, STD	1600	1612	–	–	**7.1 ± 0.2**	–	–	–
44	**γ-eudesmol**	1209-71-8	MS, RI, STD	1627	1626	–	**5.2 ± 0.9**	4.3 ± 0.1	–	–	–
45	agarospirol	1460-73-7	MS, RI	1642	1641	–	1.6 ± 1.1	0.7 ± 0.1	–	–	–
46	ζ-cadinol	5937-11-1	MS, RI	1639	1645	Tr	tr	2.0 ± 0.1	tr	tr	1.7 ± 0.2
47	α-cadinol	481-34-5	MS, RI	1658	1659	–	–	–	1.2 ± 0.2	tr	2.0 ± 0.2
48	pentadecan-2-one	2345-28-0	MS, RI, STD	1696	1697	–	–	–	4.4 ± 0.0	tr	tr
49	juniper camphor	473-04-1	MS, RI	1700	1703	–	–	1.8 ± 0.2	–	–	–
50	phytol	150-86-7	MS, RI, STD	2111	2117	–	–	–	2.9 ± 0.2	tr	tr
	Monoterpene hydrocarbons (%)					85.1	70.1	15.9	0.0	0.0	0.0
	Oxygenated monoterpenes (%)					7.0	13.2	2.6	0.0	0.0	18.2
	Sesquiterpene hydrocarbons (%)					7.2	7.4	56.5	5.1	0.0	60.8
	Oxygenated sesquiterpenes (%)					0.7	9.4	25.0	9.8	0.0	14.5
	Diterpenes (%)					0.0	0.0	0.0	2.9	0.0	0.0
	Others (%)					0.0	0.0	0.0	82.2	>99.9	6.6
	Total identified					>99.9	>99.9	>99.9	>99.9	>99.9	>99.9

Identification methods: MS, comparison of mass spectra to those of PAL 600® libraries; RI, comparison of retention indices to those reported in the literature; STD, comparison of retention times and mass spectra of commercially available standards; CAS number; RI^a^, theoretical kovats indices (Pubchem and NIST); RI^b^, calculated kovats indices; Tr, trace (a compound that represents less than 0.1% of the total peak area); -, Under perception threshold.

**Table 2:** Lipoxygenase inhibitory activity. Data are expressed as the mean of triplicates.

	IC_50_ values (µg/mL) of essential oils isolated from different organs of *Zanthoxylum* species	
	*Z. mezoneurispinosum*	*Z*. *psammophilum*
Leaves	26.4 ± 0.2	28.4 ± 0.1
Trunk bark	26.2 ± 0.2	31.3 ± 0.0
Root	25.3 ± 0.2	27.6 ± 0.1
Quercetin	21.6 ± 0.1	21.6 ± 0.1
